# How Botulinum Neurotoxin Light Chain A1 Maintains Stable Association with the Intracellular Neuronal Plasma Membrane

**DOI:** 10.3390/toxins14120814

**Published:** 2022-11-22

**Authors:** Alexander P. Gardner, Joseph T. Barbieri, Sabine Pellett

**Affiliations:** 1 Microbiology and Immunology, Medical College, Wisconsin 8701 Watertown Plank Road, Milwaukee, WI 53226, USA; 2 Department of Bacteriology, Microbial Sciences Building, University of Wisconsin-Madison, 1550 Linden Dr., Madison, WI 53706, USA

**Keywords:** botulinum toxin, bacterial toxins, SNAP-25, intracellular trafficking, protein modeling

## Abstract

Botulinum neurotoxin serotype A (BoNT/A) is the most potent protein toxin for humans and is utilized as a therapy for numerous neurologic diseases. BoNT/A comprises a catalytic Light Chain (LC/A) and a Heavy Chain (HC/A) and includes eight subtypes (BoNT/A1-/A8). Previously we showed BoNT/A potency positively correlated with stable localization on the intracellular plasma membrane and identified a low homology domain (amino acids 268–357) responsible for LC/A1 stable co-localization with SNAP-25 on the plasma membrane, while LC/A3 was present in the cytosol of Neuro2A cells. In the present study, steady-state- and live-imaging of a cytosolic LC/A3 derivative (LC/A3V) engineered to contain individual structural elements of the A1 LDH showed that a 59 amino acid region (275–334) termed the MLD was sufficient to direct LC/A3V from the cytosol to the plasma membrane co-localized with SNAP-25. Informatics and experimental validation of the MLD-predicted R1 region (an α-helix, residues 275–300) and R2 region (a loop, α-helix, loop, residues 302–334) both contribute independent steps to the stable co-localization of LC/A1 with SNAP-25 on the plasma membrane of Neuro-2A cells. Understanding how these structural elements contribute to the overall association of LC/A1 on the plasma membrane may identify the molecular basis for the LC contribution of BoNT/A1 to high potency.

## 1. Introduction

Botulinum neurotoxin (BoNT), the most potent toxin known to humans with an estimated lethal dose (LD_50_) of 1 ng/kilogram of body weight, are a family of protein toxins produced by several *Clostridia* species [1,2]. Seven immunologically distinct BoNT serotypes exist, A–G [3,4], and some *Clostridia* carry two or even three *bont* serotypes or possess a silent *bont* [5]. *Bont* gene clusters also vary among serotypes and subtypes of *Clostridia*, indicating that the entire gene clusters have undergone horizontal gene transfer and recombination, leading to diverse protein toxins [6]. In addition, several non-clostridial bacteria produce BoNT-like proteins, which share overall sequence similarity with the BoNTs [7,8,9,10,11]. 

BoNTs are produced as 150-kDa single-chain proteins that are post-translationally cleaved into disulfide-linked di-chains by a host or endogenous proteases that are classic AB-type bacterial toxins [12,13,14,15]. Cleavage is essential for maximal BoNT potency. For example, di-chain BoNT/E has been reported to be ~100-fold more toxic than single-chain BoNT/E [16]. The BoNT chains consist of an N-terminal ~50 kDa catalytic A subunit, Light Chain (LC) [17], and a C-terminal ~100 kDa host-receptor binding/translocation B subunit, Heavy Chain (HC) [18]. BoNT LCs are zinc-metalloproteases containing an (H-E-X-X-H) motif for zinc coordination and cleave neuronal Soluble N-ethylmaleimide-sensitive factor Attachment protein REceptors (SNARE) at unique sites [3]. BoNT HC possesses two domains, the translocation domain (H_N_) and the receptor binding domain (H_C_) [19]. The ~80-N-terminal amino acids of the H_N_, termed the belt, wrap around the LC within the catalytic groove and are hypothesized to maintain LC inactive until dissociated from HC [20,21]. 

BoNT/A entry into neurons follows a multi-step process. First, BoNT-H_C_ binds a polysialoganglioside, such as GT1b, on the surface of resting neurons [22], followed by binding to the BoNT/A protein receptor, Synaptic Vesicle Protein-2 (SV2), which is exposed on the surface of the depolarized plasma membrane after synaptic vesicle (SV) fusion to the plasma membrane [23]. Upon SV recycling, surface-bound BoNT is sequestered within the SV lumen [24]. As the SV matures, the lumen of the SV acidifies as the Na^+^–K^+^ ATPase pump reloads neurotransmitters with a proton into the lumen [25]. Acidification of the SV lumen to pH of 4–5 triggers H_N_ to embed into the membrane of the SV, forming a pore ~15 Å in diameter [26], which allows the unfolded LC to translocate through the H_N_ into the cytosol [27]. How LC translocates through the membrane is elusive but may follow the tunnel- or cleft-model for protein translocation [28], and translocation may be initiated at the LC C terminus [29]. After translocation, the LC-H_N_ interchain disulfide is reduced via the thioredoxin reductase–thioredoxin system, followed by LC refolding aided by HSP-90 and H_N_ [30,31]. BoNT/A LC (LC/A) then cleaves the substrate SyNaptosomal Associated Protein of 25 kDa (SNAP-25) [32] on the intracellular face of the plasma membrane. While previous studies have demonstrated LC/A1 localizes to the plasma membrane [33,34,35], the molecular mechanisms of intracellular LC trafficking to the membrane-bound SNAP-25 have not yet been elucidated. Deciphering molecular interactions involved in intracellular BoNT LC trafficking is fundamental to our understanding of the neuronal cell intoxication pathways and pathology caused by these toxins.

Several BoNT serotypes include genetic variants, which are currently defined as a new subtype denoted by numbers after the serotype letter if they differ by >2.3% at the primary amino acid level [36]. These BoNT subtypes retain sensitivity to serotype-specific antisera, and some subtypes possess unique biological activity. For example, BoNT/A comprises eight subtypes, A1–A8 [37,38], where BoNT/A1 possesses high potency and a long duration of action, while BoNT/A4 shows low potency, and BoNT/A3 has a short duration of action [39]. 

In a previous study, we observed predominant cytosolic localization of LC/A3 and determined the N terminus of LC/A and a region of low homology (LHD) between LC/A1 and LC/A3 traffic LC/A1 to the plasma membrane [40]. These studies also characterized an LC/A3 variant (LC/A3V) as a cytosolic protein. LC/A3V proved to be a platform to resolve the role of the N terminus as a facilitator of LC/A1-intracellular vesicle interactions and the LHD as a facilitator of LC/A-plasma membrane interactions. Overall, the N-terminal- and LHD-mediated interactions were independent, sequential, and additive for the movement of LC/A1 from the cytosol to the plasma membrane [35].

Since BoNT/A potency correlates with LC trafficking along vesicles and association with the plasma membrane [35,41], we have further characterized LC/A1 localization with the plasma membrane in this study. Initial experiments localized the membrane targeting capacity to the Low Homology Domain of LC/A1 (amino acids 268–357), which was divided into three structural regions (R1–R3) based upon a co-crystal of LC/A1 bound to SNAP-25 (PDB: 1XTG) [42]. Steady-state- and live time-lapse-imaging showed that while individual A1 R regions did not direct LC/A3V to the plasma membrane, the combined A1 R1:R2 regions, termed the Membrane Localization Domain (MLD) efficiently targeted LC/A3V to the plasma membrane, which involved interactions with SNAP-25. The current study used informatics and experimental validation to resolve how regions R1 and R2 are required to co-localize BoNT/A1 stably with SNAP-25 on the plasma membrane of Neuro-2A (N2A) cells, enabling the development of a molecular model for LC/A localization.

## 2. Results

### 2.1. Structural Analysis of the Low Homology Domain of LC/A1 Reveals Three Distinct Structural Regions

**Figure 1:**
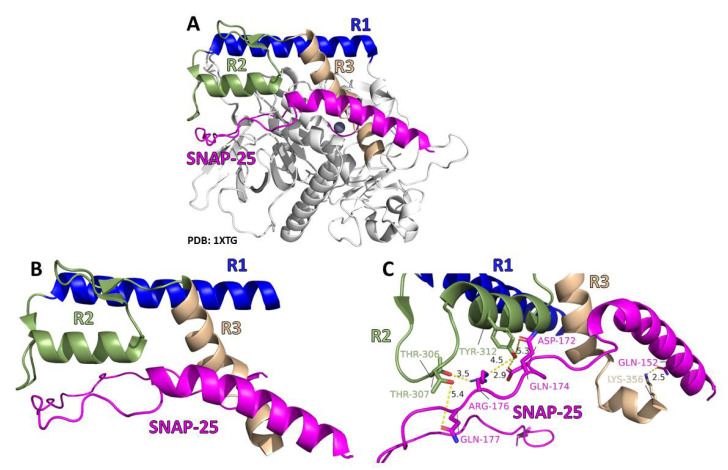
Co-crystal structure of the Botulinum neurotoxin LC/A1 and SNAP-25 (PDB: 1XTG). (**A**) The structure of LC/A1 is gray with regions of the LHD colored, with R1 in blue, R2 in smudge green, R3 in wheat, and SNAP-25 in magenta. (**B**) Enhancement of the R1:R2:R3 with SNAP-25, with R1 in blue, R2 in smudge green, R3 in wheat, and SNAP-25 in magenta. (**C**) Distances between potential non-covalent interactions were measured with PyMOL. R2 of LC/A1 is highlighted in smudge green, R3 of LC/A1 is highlighted in wheat, and SNAP-25 is highlighted in magenta.

The current study sought to establish how the previously identified LHD (268–357) mediated LC/A1 association with the plasma membrane [35]. A co-crystal of LC/A1 bound to SNAP-25 (residues 146–204 (PDB: 1XTG)) and the recently solved crystal structure of LC/A3 [43] showed the LHD to be organized into three sequential structural regions (R1–R3 (Figure 1). R1, residues 275–300, included an α-helix; R2, residues 302–334, included a loop, α-helix, and loop; and R3, residues 335–357, included an α-helix, loop, and α-helix. Assessment of the LC/A1-SNAP-25 co-crystal showed R1 was distanced from bound SNAP-25, R2 had ionic and hydrogen bonds with bound SNAP-25, and R3 had a single ionic bond bound to SNAP-25 [42]. Next, we determined if R1, R2, and R3 contributed to the stable association of LC/A1 on the inner plasma membrane of N2A cells [35,40].

### 2.2. Regions R1 and R2 of the A1 LHD Target LC/A3V to the Plasma Membrane 

LC/A3V is a cytosolic protein when ectopically expressed in N2A cells, and replacing the A3 LHD with A1 LHD transitioned LC/A3V from the cytosol to the plasma membrane [35]. Here, LC/A3V was constructed, in which individual (R1, R2, or R3) or dual (R1:R2, R2:R3, or R1:R3) regions of the LHD with the corresponding A1 region and fusion proteins with Enhanced Green Fluorescent Protein (EGFP)-LC/A3V(A1 R) were expressed in N2A cells (Table 1). Cells were imaged to measure the transition from the cytosol to the plasma membrane (Supplemental Figure S1). Western blotting showed that each LC/A3V(A1 R) chimera was expressed in N2A cells with the appropriate molecular weight and at similar expression levels (Supplemental Figure S2).

**Figure 2:**
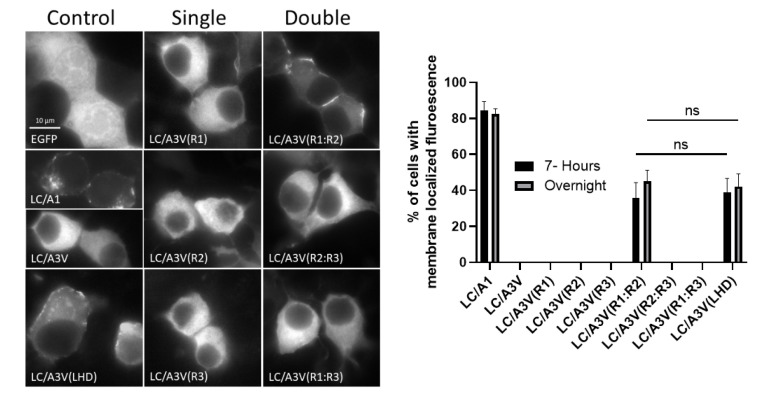
Intracellular localization of GFP-LC/A3V (A1-LHD) chimeras. (**Left**) After overnight transfections with EGFP-LC/A derivatives and fixation with 4% paraformaldehyde, N2A cells were imaged for EGFP fluorescence (excitation 488 nm, emission 509 nm). Representative images show the steady-state intracellular localization of CONTROL (EGFP, EGFP-LC/A1, EGFP-LC/A3V), SINGLE (EGFP-LC/A3V (A1 R1), EGFP-LC/A3V (A1 R2), EGFP-LC/A3V (A1 R3)), or DOUBLE (EGFP-LC/A3V (A1 R1:R2), EGFP-LC/A3V (A1 R2:R3), EGFP-LC/A3V (A1 R1:R3)), and EGFP-LC/A3V (A1- LHD). (**Right**) Percentage of cells containing membrane signal for 7 h post-transfection and overnight. Ten random fields were selected and counted for membrane localization with wheat germ agglutinin. Mean and SEM were evaluated as described previously [35]; ns—not significant.

At steady-state, individual A1 R regions did not transition cytosolic LC/A3V from the cytosol to the plasma membrane (Figure 2). In contrast, the dual-A1 R region chimera targeted LC/A3V(R1:R2) to the plasma membrane, while LC/A3V(R2:R3) and LC/A3V(R1:R3) were expressed in the cytosol. Thus, A1 R1:R2 was necessary and sufficient to target LC/A3V to the plasma membrane.

**Figure 3:**
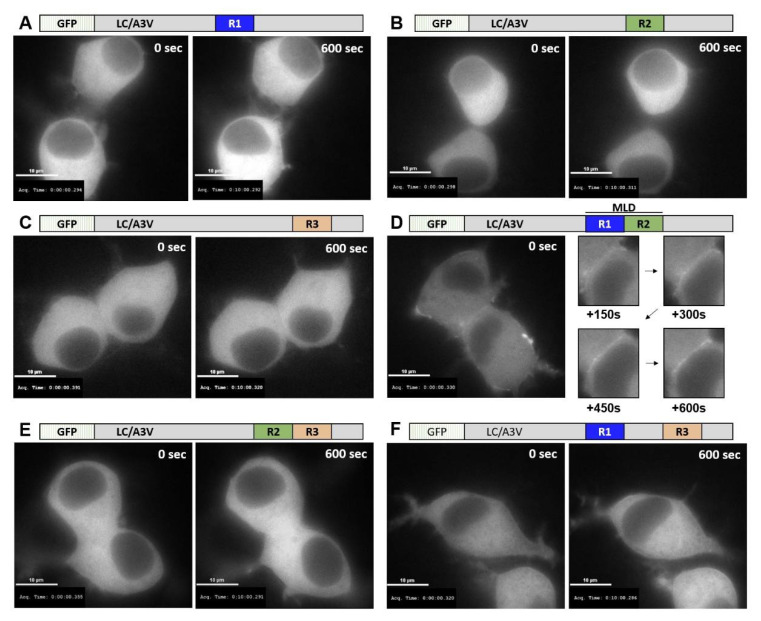
Time-lapse imaging of GFP-LC/A3V (A1-LHD) chimeras. After a seven-hour transfection with plasmids encoding (**A**) EGFP-LC/A3V (A1 R1), (**B**) EGFP-LC/A3V (A1 R2), (**C**) EGFP-LC/A3V (A1 R3), (**D**) EGFP-LC/A3V (A1 R1:R2) or EGFP-LC/A3V [MLD], (**E**) EGFP-LC/A3V (A1 R2:R3), and (**F**) EGFP-LC/A3V (A1 R1:R3) were obtained as indicated. N2A cells were imaged every ten seconds for ten minutes on a Nikon Eclipse Ti-inverted microscope, using a 60× oil-immersion objective (1.4 NA objective) hardware with Eclipse software for data analysis. Images are from the initial image +0 s and the final image +600 s unless otherwise specified.

At 7 h post-transfection, when EGFP fluorescence was initially detected, LC/A3V(A1 R) chimeras showed similar localization as observed for steady-state expression; only LC/A3V(R1:R2) localized on the plasma membrane (Figure 2). Live imaging at 7 h post-transfection also showed a stable accumulation of LC/A3V(R1:R2) on the plasma membrane directly from the cytosol with no observed membrane dissociation over a 10 min measurement (Figure 3). Thus, the exchange of only A1 R1:R2 into LC/A3V was necessary and sufficient to target LC/AV3 from the cytosol to the plasma membrane, similar to LC/A3V(A1 LHD) described earlier [35]. We also noted that the association of LC/A3V(R1:R2) was stable on the plasma membrane. We termed this newly defined region, which spans amino acids 275–334, the membrane localization domain (MLD).

### 2.3. The A1 MLD and N Terminus Have Additive Functions in the Transition of LC to Vesicles, and the Plasma Membrane

Earlier studies showed that the N terminus and the LHD of LC/A1 contribute in an additive manner to target LC/A3V from the cytosol to vesicles and then to the plasma membrane, respectively [35].

**Figure 4:**
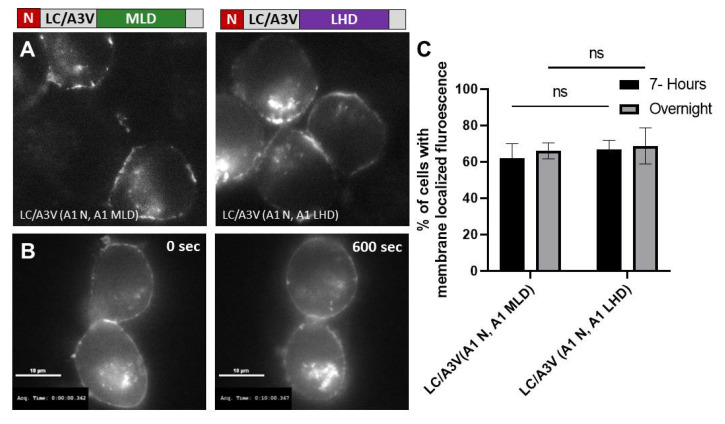
A1 MLD retains the LHD function. (**A**) Representative images show the steady-state intracellular localization of EGFP-A3V(A1 N, A1 MLD) residues 1–17 and 275–334 and EGFP-A3V(A1 N, A1 LHD) residues 1–17 and 268–357, indicated with a cartoon schematic. (**B**) After a seven-hour transfection, EGFP-A3V(A1 N, MLD). Live cell images were obtained every 10 s for 600 s; the initial frame (0 s) and final frame (600 s) are shown. (**C**) Percentage of cells containing membrane fluorescence for 7 h post-transfection and overnight were analyzed. Mean and SEM were evaluated as described previously [35]; ns—not significant.

Therefore, the A1 MLD was tested as a minimal substitute for the A1 LHD to coordinate the transition of LC/A1 from vesicles to the plasma membrane. Utilizing the cytosolic LC/A3V platform, EGFP-LC/A3 fusion proteins were created, replacing the MLD and 17 aa N-terminal regions with the corresponding regions of A1, and expressed in N2A cells. At 7 h post-transfection, time-lapse imaging showed LC/A3V(A1 N, A1 MLD) associated with intracellular vesicles with stable accumulation on the plasma membrane (Figure 4) [35]. Similar to the A1 LHD, A1 MLD coordinated with the N terminus to anterograde traffic LC/A3V from the cytosol to the plasma membrane.

### 2.4. Role of A1 R1:R2 in MLD Transition of LC/A3V from the Cytosol to the Plasma Membrane

**Figure 5:**
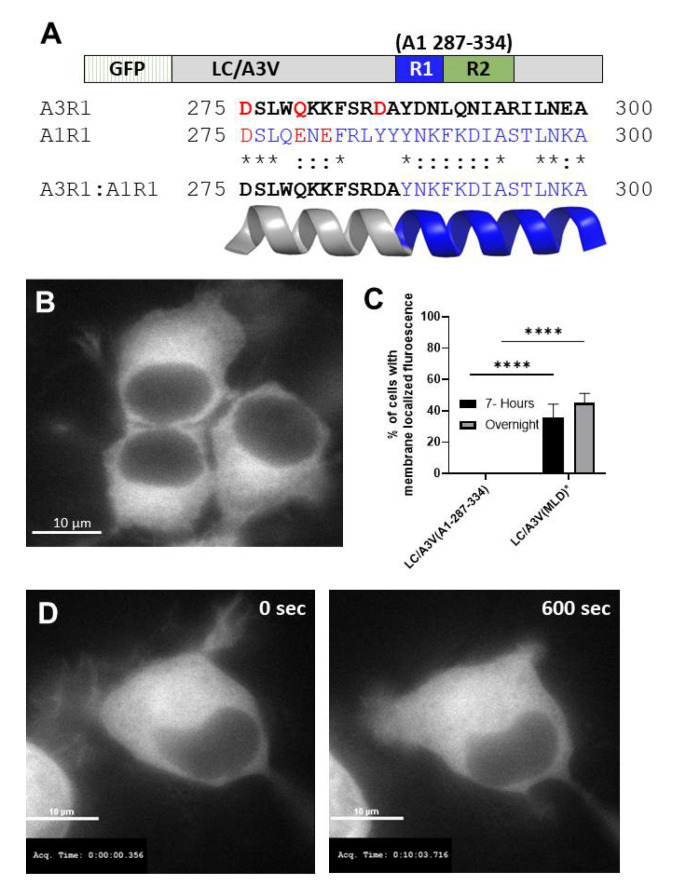
The N terminus of the R1 region of the MLD is necessary for LC/A1 membrane localization. (**A**) The crystal structure of LC/A1 (PDB: 1XTG) and the crystal structure of LC/A3LM (PDB: 7DVL) were analyzed for amino acid differences. Residues 275–300 comprise a surface-exposed α-helix. The primary amino acid sequences of A3LM (top) ACA57525 and A1 (bottom) ACS66881were analyzed by Blastp. Below, A3R1 and A1R1 depict identical amino acids between A3 LM and A1 (*); conserved amino acids (:); and non-conserved amino acids ( ). The bottom line is the A3R1:A1R1 chimera sequence. (**B**) A representative image shows the steady-state intracellular localization of EGFP-A3V(A1-287–334). (**C**) The percentage of cells containing membrane fluorescence at 7 h post-transfection and overnight were analyzed. Mean and SEM were evaluated as described previously [35]; **** *p* < 0.0001. (**D**) After a 7 h transfection, EGFP-A3V(A1-287–334) was imaged as indicated above. Live cell images were obtained every 10 s for 600 s. Images are from the initial frame (0 s) and the final frame (600 s).

Properties of R1. Sequence alignment showed that the N-terminal residues 275–286 of A1-R1 are polar and acidic, while A3-R1 residues are polar and basic (Figure 5A) [43]. Thus, a chimera containing the N terminus of A3-R1 fused to the C terminus of A1-R1 (A1-287–334) was engineered to investigate the contribution of the N-terminal region of A1-R1 in targeting LC/A3V from the cytosol to the plasma membrane. At 7 h- and steady-state-post-transfection, LC/A3V(A1-287–334) was cytosolic, analogous to LC/A3V (Figure 5B,C). Time-lapse imaging 7 h post-transfection showed LC/A3V(A1-287–334) retained a cytosolic phenotype with no detected temporal accumulation at the plasma membrane (Figure 5D). Together, the steady-state and time-lapse imaging indicate that A1-R1 (residues 275–286) is necessary to localize LC/A3V to the plasma membrane.

**Figure 6:**
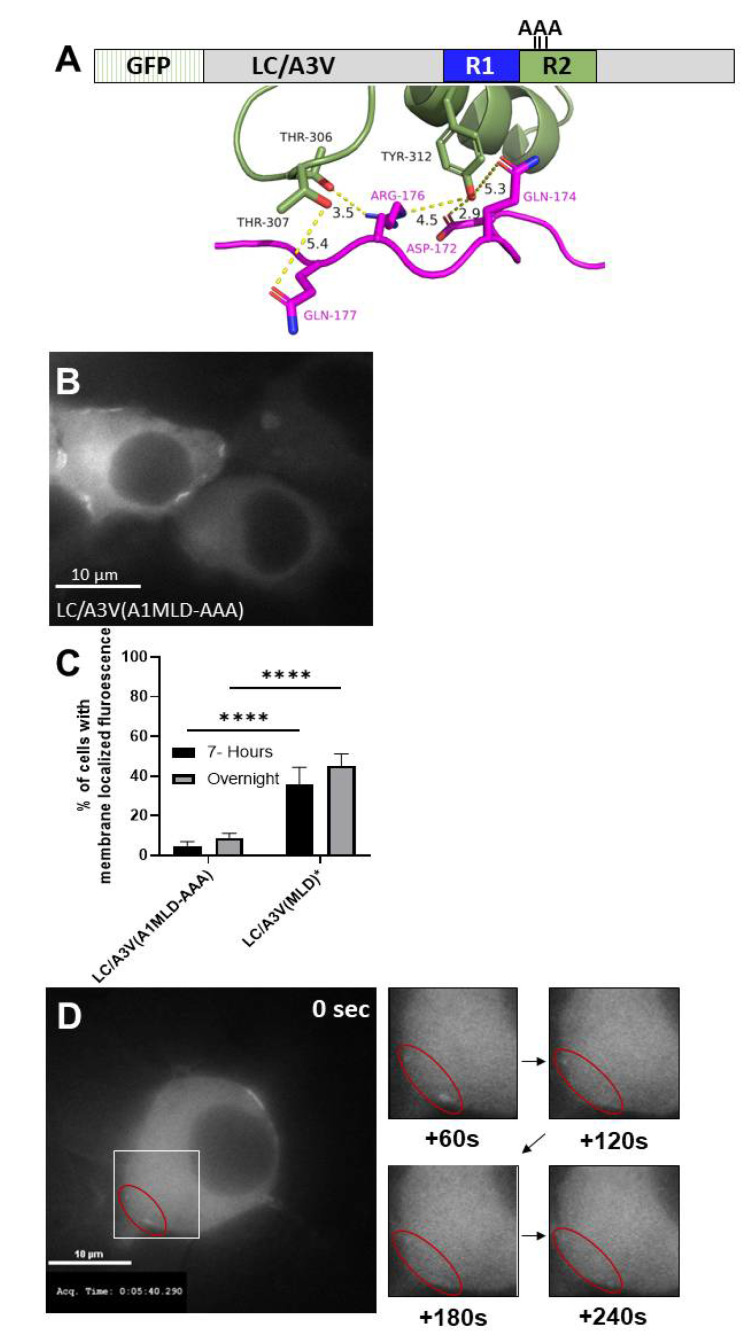
MLD-SNAP-25 interaction within the R2 is necessary for stable plasma membrane association of LC/A1. (**A**) The LC/A1-SNAP-25 co-crystal crystal structure (PDB: 1XTG) was analyzed for possible interactions. The distances between potential non-covalent interactions were measured with PyMOL. R2 of LC/A1 is highlighted in smudge green, with SNAP-25 highlighted in magenta. (**B**) The representative image shows the steady-state intracellular localization of EGFP-A3V(A1 MLD) AAA. (**C**) Percentage of cells containing membrane fluorescence for 7 h post-transfection and overnight were analyzed. Mean and SEM were evaluated as described previously [35]; **** *p* < 0.0001. (**D**) After a seven-hour transfection, EGFP-A3V(A1MLD-AAA) was imaged as indicated above. Red circles indicate membrane localized EGFP-LC/A3V(A1MLD-AAA) that diffused into the cytosol over 240 s. Live cell images were obtained every 10 s for 600 s; the indicated time frames are shown.

Properties of R2. Using the LC/A1-SNAP-25 co-crystal structure (PDB: 1XTG) (Figure 6A), R2 residue Y312 was reported to bond with SNAP-25 D172, N174, and R176 [42,43]. Further examination of the LC/A1-SNAP-25 co-crystal structure also predicted two additional R2 residues, T306 and T307, to bind SNAP-25 R176 and Q177, respectively (Figure 6A). Site-directed mutagenesis of T306, T307, and Y312 to A resulted in a new chimera (MLD-A306A307A312, termed MLD-AAA). At both 7 h- and steady-state-post-transfection, LC/A3V(A1MLD-AAA) was expressed as a cytosolic protein in all transfected cells. A subpopulation of transfected cells ~8%, LC/A3V(A1MLD-AAA) that were cytosolically expressed also possessed detectable localization on the plasma membrane, indicating T306, T307, and Y312 to A306, A307, and A312 resulted in an intermediate phenotype (Figure 6B,C). Time-lapse imaging showed both the movement of LC/A3V(A1MLD-AAA) off the plasma membrane, indicating that LC/A3V(A1MLD-AAA) was less stable on the plasma membrane than LC/A3V(A1MLD) with the diffusion of LC/A3V(A1MLD-AAA) into the cytosol during the 10 min exposure (Figure 6D, time lapse images) and stable plasma membrane bound LC/A3V(A1MLD-AAA) (Figure 6D 0 sec, upper right corner of cell). These findings showed that alanine mutations at T306, T307, and Y312 transitioned LC/A3V(A1MLD-AAA) to the cytosol, with ~8% also bound either stably or reversibly on the plasma membrane relative to the stable membrane association of LC/A3V(A1MLD), implicating a role for A1R2 in the interaction with SNAP-25 on the plasma membrane.

### 2.5. The LC MLD and Membrane Localization Are Conserved amongst BoNT/A Subtypes Other Than A3

**Figure 7:**
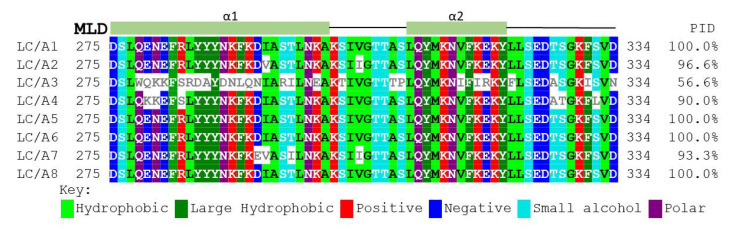
Clustal Omega alignment of BoNT/A subtypes residues 275–334. Alignment of LC/A1 ACS66881, LC/A2 CAA51824, LC/A3 ACA57525, LC/A4 ACQ51417, LC/A5 ACG50065, LC/A6 ACW83608, LC/A7 AFV13854, and LC/A8 AJA05787 were aligned using Clustal Omega [44]. Number and secondary structure elements (green α-helix) are shown for the LC/A subtypes. Percent identity (PID) was determined off the LC/A1 sequence. The image was adapted by the MView tool [45]. Background indicates conserved residue, and the color schematic corresponds to the side chain properties; the key is below the sequence.

**Figure 8:**
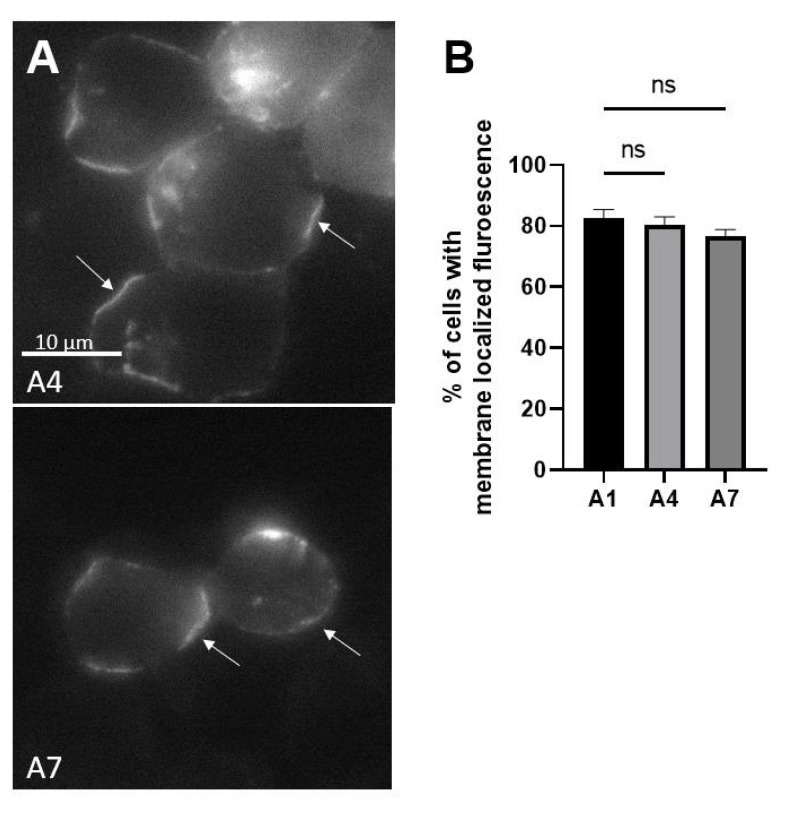
Intracellular localization of LC/A4 and LC/A7. N2A cells were transfected with pEGFP-LC/A4 or LC/A7. After overnight transfections, N2A cells were fixed with 4% paraformaldehyde and imaged for EGFP fluorescence (excitation 488 nm, emission 509 nm). (**A**) A Representative image shows the steady-state intracellular localization of EGFP-LC/A4 and EGFP-LC/A7, with white arrows indicating membrane localization. (**B**) Percentage of cells containing membrane fluorescence. Ten random fields were selected and counted for membrane localization. Mean and SEM were evaluated as described previously [35]; ns = no significant statistical significance determined between indicated transfected cells.

Clustal alignment of BoNT/A(A1–A8) showed the MLDs of BoNT/A1, /A5, /A6, and /A8 MLD were identical (Figure 7). The two amino acid differences, V^293^ and I^304^, of BoNT/A2 compared to A1 do not influence intracellular localization since LC/A2 localizes on the plasma membrane [35]. The MLD of BoNT/A4 had six amino acid changes relative to BoNT/A1 at R1 (K^279^, K^280^, S^283^) and R2 (A^327^, T^328^, and L^332^). Despite these six amino acid changes, EGFP-LC/A4 was expressed in N2As primarily as a plasma membrane-localized protein (Figure 8), indicating these residues do not contribute to membrane localization. The MLD of BoNT/A7 had four residue changes relative to BoNT/A1 in R1: E^292^, V^293^, I^296^, and in R2 I^304^. Despite these four amino acid changes, EGFP-LC/A7 was expressed in N2As primarily as a plasma membrane-localized protein (Figure 8), indicating these residues do not contribute to membrane localization. Extrapolation of the data on LC/A1, /A2, /A4, and /A7 predicts LC/A5, /A6, and /A8 will localize at the plasma membrane, as the MLDs are identical to LC/A1. Thus, among the BoNT/A subtypes, only BoNT/A3 shows limited membrane localization [35]. 

## 3. Discussion

**Figure 9:**
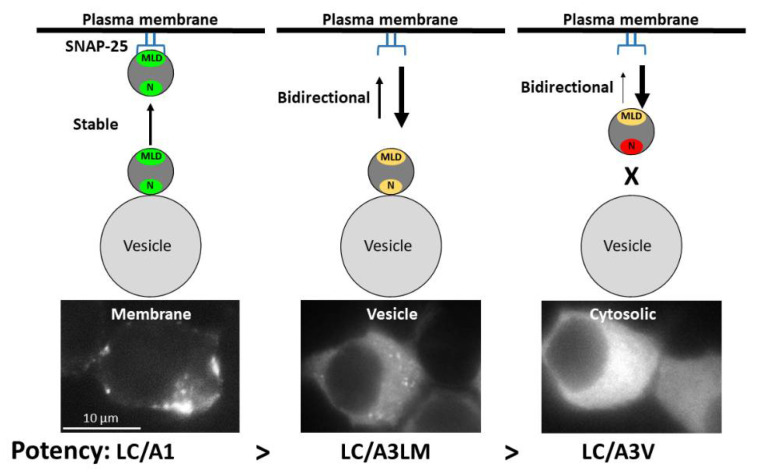
Intracellular localization of BoNT/A subtypes- LC/A1, LC/A3LM, and LC/A3V. The N terminus (green N) associates LC/A1 and (yellow N) LC/A3LM with a synaptic vesicle, while the N terminus (red N) fails to associate LC/A3V with synaptic vesicles. LC/A1 MLD (green MLD) possesses a high affinity for localization at the plasma membrane where the (yellow MLD) LC/A3LM or (yellow MLD) LC/A3V fails to localize to the plasma membrane stably. LC/A3V can anterograde traffic (diffusion) to the plasma membrane but does not stably associate with the plasma membrane, while LC/A3LM can traffic to the plasma membrane but does not stably associate with the plasma membrane, leading to the three phenotypes of the respective LCs imaged.

Earlier studies showed the LHD (residues 268–357) of BoNT/A1 was necessary for stable LC/A1 localization at the plasma membrane [35]. The current study’s structural analysis of LC/A1-SNAP-25 co-crystal (PDB: 1XTG) revealed that the LHD domain comprised three regions: R1, which was physically separated from SNAP-25; R2, which contributed to the SNAP-25 binding pocket; and R3, which was juxtaposed to a SNAP-25 α-helix. Utilizing EGFP-LC/A3V as a reporter platform [35], R1–R2, residues 275–334, termed the membrane localization domain (MLD), targeted EGFP-LC/A3V stably to the intracellular plasma membrane of N2A cells at 7 h and overnight steady-state expression (Figure 2). The 7 h post-transfection was investigated to observe if any EGFP-LC/A overexpression occurred at steady-state, leading to false-positive localization. As previously observed [35], adding the LC/A1 N terminus, residues 1–17, facilitated the transition to the plasma membrane (Figure 4). Loss of membrane localization for LC/A3V(A1R1) or LC/A3V(A1R2) supported roles for R1 and R2 to mediate a stable association with SNAP-25 (Figure 2). Interestingly, R1 may mimic another SNAP-25 binding protein, rabphilin-3a, targeting the N terminus of SNAP-25; we propose that this association, together with the association of R2 with the C terminus of SNAP-25, combine to mediate the stable co-localization with SNAP-25. These findings support the sequential step model for the intracellular localization of LC/A1 described in [35], where the N terminus of LC/A1 targets LC anterograde trafficking on vesicles, and the MLD targets LC/A1 to stably co-localize with SNAP-25 on the plasma membrane (Figure 9). 

SNAP-25, a member of the SNARE family, is essential for exocytosis within neuronal cells. SNARE proteins are classified into five subfamilies depending on the structure, with SNAP-25 classified as a Qbc-SNARE [46]. While most SNARE proteins possess a transmembrane domain, SNAP-25 is a soluble protein that requires palmitoylation for association with the plasma membrane [47]. Before palmitoylation, SNAP-25 interacts with syntaxin-1, which is hypothesized to mediate the initial membrane binding of SNAP-25 [48,49]. After priming and uncoupling, SNAP-25 recycles to the trans-Golgi network and recycling endosomes before trafficking back to the plasma membrane [50]. While recycling back to the plasma membrane, SNAP-25 and LC/A1 remain segregated and populated on different vesicles, while LC/A1 co-localized with plasma membrane-associated SNAP-25 (Supplemental Figure S3), suggesting that the trafficking of LC/A1 to the plasma membrane mediated by the N terminal-17 amino acids of LC/A1 is uncoupled from SNAP-25 cycling to the plasma membrane. Our data support the current models for the trafficking pathway of SNAP-25. 

Clustal alignment showed 100% identity for the MLDs of A1, A5, A6, and A8. LC/A2 was previously shown to localize with the plasma membrane despite having several conserved differences in the MLD relative to LC/A1 (Figure 7). LC/A4 and LC/A7 presented as membrane-localized proteins (Figure 8), demonstrating that despite amino acid differences within the MLD, residues that contribute to membrane localization are conserved. In contrast, the LC/A3 WT variant LC/A3 Loch Maree (LC/A3LM), which localizes on SV and the cytosol, has the lowest homology, 56.6% within the MLD relative to LC/A1 [35]. Our earlier data correlate with BoNT/A potency and intracellular localization, where A3LM and A3V have consecutively lower potency than A1 and increased cytosolic presence (Figure 9) [35,51]. 

The average duration of a eukaryotic protein is 90 min to 48 h [52,53,54]. Proteins with longer half-lives are classified as long-lived proteins (LLPs); these LLPs have been identified within synaptosomes and protein complexes [55]. In non-dividing cells such as neurons, components of the nuclear pore complex and some histones are preserved for years, as neurons within brains are hypothesized not to turn over [56,57,58]. Analyzing LLPs correlates membrane localization to protein duration, as cytosolic forms of the LLPs have a higher turnover rate than the respective plasma membrane LLP derivative [55]. The differences in localization suggest that cytosolic proteins are more accessible to the degradation machinery, resulting in a higher turnover. In addition to localization, two more factors have been shown to contribute to protein turnover: the primary sequence and incorporation into protein complexes or cellular structures [55]. The molecular basis for stable interactions between LLPs and intracellular membranes remains to be determined. Data show that in primary rodent neurons, the duration of action, a function of the LC, of BoNT/A1, BoNT/A2, and BoNT/A4 is >9 months, while that of BoNT/A3LM is <5 months [40,59]. Additionally, analysis of BoNT/A in a mouse model of botulism [39] showed a rapid recovery upon local intoxication with BoNT/A3 compared to BoNT/A1 and BoNT/A2. Analyzing the intracellular localization of the BoNT/A LCs: A1, A2, and A4 are plasma membrane-localized and co-localized with SNAP-25 [33,34,35] (Figure 8), while LC/A3LM is cytosolic and vesicle based [35]. Another protein interaction of LC/A1 that participates in stabilization may be the previously observed interaction of the C terminus, a deubiquitinating enzyme VCIP-135, which removes ubiquitin [60]. Therefore, intracellular localization and protein–protein interactions of BoNT/A LCs may be associated with duration of action, as subtypes with long durations, namely, A1, A2, A4, and A7, are membrane-localized. In contrast, shorter durations correlate with non-plasma membrane-localized LCs, such as LC/A3LM [39] and LC/E [61]. 

## 4. Conclusions

The current study provides novel insight into the mechanism behind the stable intracellular localization of LC/A1 in N2A cells, a model for primary motor neurons, based upon differences in the primary amino acid sequences of the LC/A subtypes, despite a conserved secondary amino acid structure. This level of refinement to identify unique host protein–BoNT interactions reveals new details that will aid in developing novel BoNT therapeutics and mechanisms targeting the duration of action of BoNTs. 

## 5. Materials and Methods

### 5.1. Light Chain Structural Alignment 

Co-crystal structures of BoNT LC/A1 bound to SNAP-25 (PDB: 1XTG) and LC/A3 Loch Maree (LM) (PDB: 7DVL) were obtained from the protein data bank (https://www.rcsb.org (14 January 2021)) and aligned using PyMol (PyMOL Molecular Graphic System, Version 2.0 Schödinger, LLC. (New York, NY, USA)). Images were enhanced using the “ray” trace command and exported as a PNG file. 

### 5.2. BoNT-LC/A1 and /A3V Sequence Alignment 

BoNT/A1 (ACS66881) and BoNT/A3 (ABY56337) were obtained from Uniprot [62] and aligned using the Blastp Suite (USS National Library of Medicine) [63] to identify residues within regions of interest within the low homology domain (LHD) residues 268–357. Regions within the LC/A1 LHD were assigned: R1 (275–300), R2 (302–334), and R3 (335–357) (Supplemental Figure S4).

### 5.3. Engineering of GFP-LC/A3V (A1 LHD) Chimera Expression Plasmids

DNA encoding LC/A1 (1–450) and LC/A3V (1–446) were engineered as enhanced green fluorescent protein (EGFP)-fusions [35] within pEGFP-C3 by subcloning the LC genes into the SacI-BamHI restriction sites. New England Biolabs^®^ NEBaseChanger^®^ was used to design primers to engineer the chimeras EGFP-LC/A3V-A1, EGFP-LC/A3V(A1 275–300), EGFP-LC/A3V(A1 302–334), EGFP-LC/A3V(A1 335–357), EGFP-LC/A3V(A1 275–334), EGFP-LC/A3V(A1 302–357), EGFP-LC/A3V(A1 275–300, 335–357), EGFP-LC/A3V (A1 268–357), EGFP-LC/A3V (A1 1–17, 275–334), EGFP-LC/A3V (A1 287–334), and EGFP-LC/A3V (A1 275–334) T^306^A, T^307^A, Y^312^A (Table 1). 

### 5.4. Expression, Capture, and Analysis of Immunofluorescent EGFP-LC/A Plasmids

EGFP-LC/A expression plasmids were transfected into Neuro-2A (N2A) cells and scored following the protocol previously described [35]. Cells were plated onto 24-well plates (Fisher Scientific, Chicago, IL, USA) at a seeding density of 50,000 cells/well in complete essential media supplemented with 10% fetal bovine serum, 1× penicillin–streptomycin, 0.1% sodium bicarbonate, 1 mM sodium pyruvate, and 1% nonessential amino acids in humidified 5% CO_2_ at 37 °C. The following day, N2A cells were transfected as described by the manufacturer (Lipofectamine LTX; Invitrogen™ (Waltham, MA, USA)) with 500 ng of the indicated plasmid in antibiotic-free complete essential media supplemented with 10% fetal bovine serum, 0.1% sodium bicarbonate, 1 mM sodium pyruvate, and 1% nonessential amino acids. Following a 7 h or overnight incubation, N2A cells were subjected to a membrane stain, wheat germ agglutinin (WGA): Alexa Fluor^647^ (1:1000), for 30 min at 4 °C. N2A cells were fixed with 4% paraformaldehyde for 15 min prior to staining with a nuclear marker, Hoechst (1:10,000), for five minutes and mounting to a glass coverslip with 8 µL of Prolong™ Gold Antifade Mountant (Invitrogen, Waltham, MA, USA). After staining, N2A cells were imaged on a Nikon Eclipse Ti-inverted microscope, using a 60× oil-immersion objective (1.4 NA objective) hardware with Eclipse software for data analysis. For each transfection condition, ten random fields of N2A cells were scored for EGFP localization for a total of ~100 N2A cells, as previously described [35]. EGFP was scored membrane-bound when fluorescence co-localized with wheat germ agglutinin. EGFP empty vector co-localized in the cytosol and with Hoechst in the nucleus. Cells positive for membrane determined by localization with wheat germ agglutinin (WGA) were scored as a % of total cells utilizing the following equation [40]: (% of cells expressing membrane localization/total number of cells with EGFP fluorescence) × 100. Immunofluorescence results were graphed utilizing GraphPad Prism 9.3.1 (San Diego, CA, USA) and subjected to a statistical test using ordinary one-way ANOVA with Dunnett’s multiple comparisons with LC/A3V (A1-LHD) as the control column. Western blotting showed that each EGFP-LC/A fusion protein expressed and migrated to the correct molecular weight as indicated by SDS-PAGE (Supplemental Figure S2).

### 5.5. Live-Cell Imaging

Cells were plated and analyzed as previously described [35,64]. Cells were plated onto a 35 mm dish (MatTek, Ashland, MA, USA) at a seeding density of 300,000 cells in complete essential media supplemented with 10% fetal bovine serum, 1× penicillin–streptomycin, 0.1% sodium bicarbonate, 1 mM sodium pyruvate, and 1% nonessential amino acids in humidified 5% CO_2_ at 37 °C. The following day, N2A cells were transfected as described by the manufacturer (Lipofectamine LTX; Invitrogen™ (Waltham, MA, USA)) with 2000 ng of the indicated plasmid in antibiotic-free complete essential media supplemented with 10% fetal bovine serum, 0.1% sodium bicarbonate, 1 mM sodium pyruvate, and 1% nonessential amino acids. Seven hours post-transfection, N2A cells were live-imaged on a Nikon Eclipse Ti-inverted microscope using a 60× oil-immersion objective (1.4 NA objective) with a heated stage at 37 °C (Frank E. Fryer A-50) with Eclipse software for data analysis. Images were acquired every ten seconds for 10 min. Videos and images were compiled utilizing Nikon Elements AR 1.60.00 64-bit software (Melville, NY, USA) and Image J [65,66]. 

### 5.6. Western Blotting Confirming Chimera Molecular Weight

After overnight transfection, N2A cells were lysed using a 2× Protein Sample Buffer (150 µL) and boiled at 100 °C for five minutes. Cell lysates were resolved to a 13.5% SDS-PAGE and transferred to an Immobilon-P polyvinylidene difluoride membrane (PVDF) (Millipore, Billerica, MA, USA). PVDF membranes were fixed with methanol, air dried, rehydrated with methanol and rinsed in H_2_O, and then incubated for 30 min in a blocking solution (2% powder milk *w*/*v* in 0.1% TBST). After blocking, PVDF membranes were probed with primary rat α-EGFP-monoclonal IgG (1:2000) (3H9, Chromotek, Planegg, Germany) for 60 min. Bound primary antibodies were recognized with α-rat IgG conjugated with horseradish peroxidase (1:10,000) (Life Technologies, Waltham, MA, USA). Secondary antibodies were visualized using Super Signal™ West Pico PLUS Chemiluminescent Substrate (34578, Thermo, Rockford, IL, USA) on an Azure C600 Imaging System (Dublin, CA, USA), using 20-second exposure.

## Figures and Tables

**Table 1:** GFP-LC/A3V (A1 R) chimeras.

LC/A3V(A1-LHD) Chimeras *	Designation	Chimera Schematic
EGFP-LC/A3V(A1 275–300)	LC/A3V(R1)	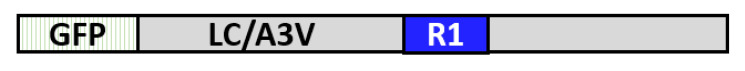
EGFP-LC/A3V(A1 302–334)	LC/A3V(R2)	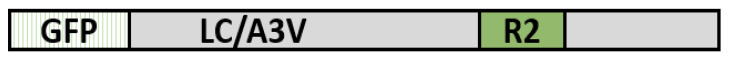
EGFP-LC/A3V(A1 335–357)	LC/A3V(R3)	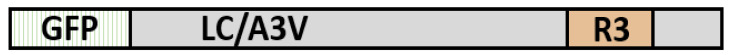
EGFP-LC/A3V(A1 275–334)	LC/A3V(R1:R2) [MLD]	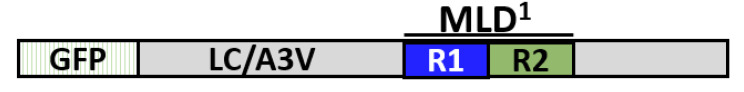
EGFP-LC/A3V(A1 302–357)	LC/A3V(R2:R3)	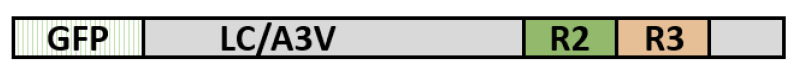
EGFP-LC/A3V(A1 275–300, 335–357)	LC/A3V(R1:R3)	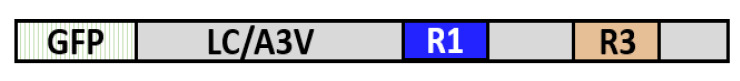
EGFP-LC/A3V (A1 268–357)	LC/A3V(LHD)	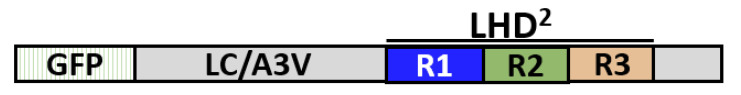
EGFP-LC/A3V (A1 1–17, 275–334)	LC/A3V(A1 N, A1 R1:R2) [MLD]	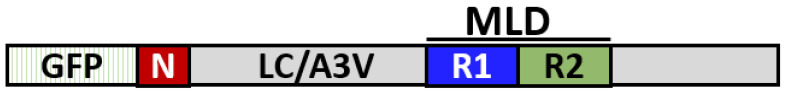
EGFP-LC/A3V (A1 287–334)	LC/A3V(A1-287-334)	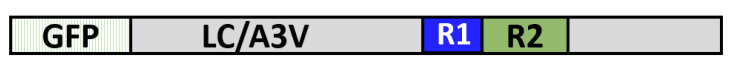
EGFP-LC/A3V (A1 275–334) T^306^A, T^307^A, Y^312^A	LC/A3V(A1MLD-AAA)	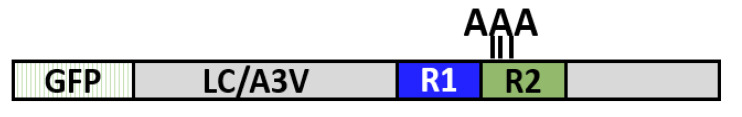

* Chimeras utilized LC/A3V (residues 1–446) as a platform, exchanging regions from LC/A1 LHD. ^1^ MLD: membrane localization domain residues 275–334 from LC/A1. ^2^ LHD: low homology domain residues 268–357 from LC/A1.

## Data Availability

The methods and data and supplementary data presented in this study are available.

## Supplementary Materials

The following supporting information can be downloaded at: https://www.mdpi.com/article/10.3390/toxins14120814/s1, Figure S1: Intracellular localization of BoNT/A LC/A1; Figure S2: Western blotting of GFP-LC/A3V (A1-LHD) chimeras; Figure S3: Z-stack series of GFP-LC/A1 and SNAP-25; Figure S4: Blastp alignment of LC/A3 LM with LC/A1.
