# Supplementary material for: How Botulinum Neurotoxin Light Chain A1 Maintains Stable Association with the Intracellular Neuronal Plasma Membrane
Source: Toxins (Basel). 2022 Nov 22;14(12):814. doi: 10.3390/toxins14120814 (PMC9783275; doi:10.3390/toxins14120814)
Supplement: Supplementary file 1 [file toxins-14-00814-s001.zip › toxins-2006280-supplementary.pdf]

toxins

MDPI

Supplementary Materials: How Botulinum Neurotoxin Light Chain A1 maintains Stable Association with the Intracellular Neuronal Plasma Membrane

Alexander P. Gardner, Joseph T. Barbieri and Sabine Pellett

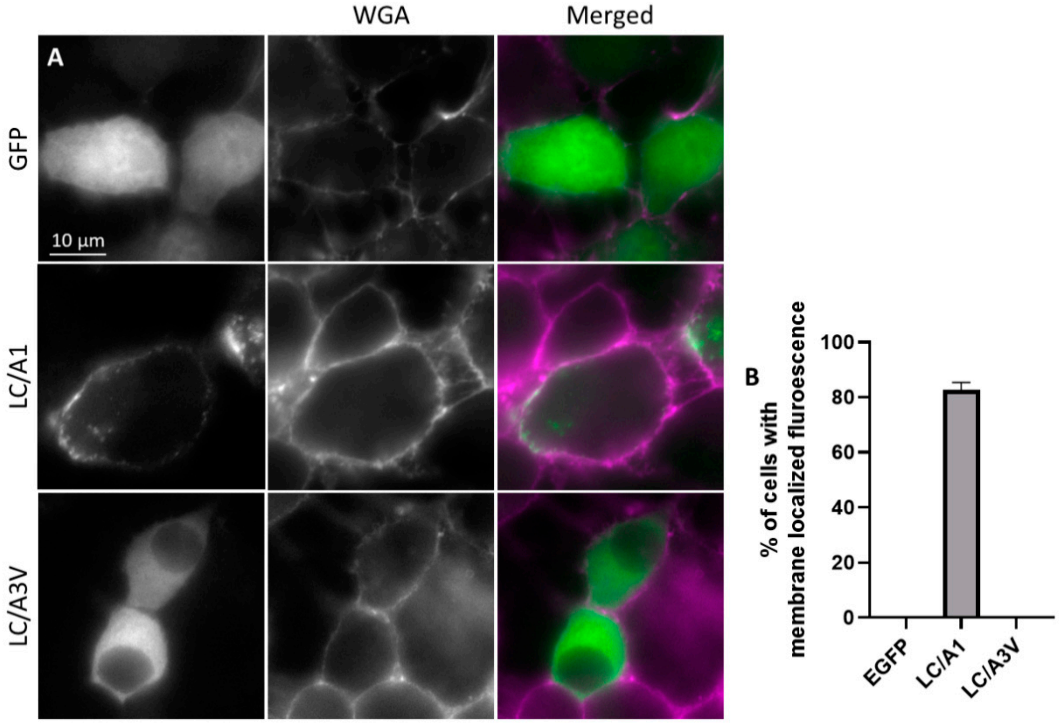

Supplemental Figure S1 Intracellular localization of BoNT/A LC/A1. (*A*) After overnight transfections with EGFP-LC/A1 and fixation with 4% paraformaldehyde, N2A cells were stained for membranes with wheat germ agglutinin (WGA) and imaged for EGFP fluorescence (excitation 488 nm, emission 509 nm) and WGA fluorescence (excitation 645 nm, emission 705 nm). Control included as cytosolic-localized EGFP-C3 Representative staining shows EGFP fluorescence and WGA staining followed by merged: EGFP-C3 was present in the cytosol and nucleus, EGFP-LC/A1 localized on the cell membrane, and EGFP-LC/A3 was present in the cytosol and absent in the nucleus. (B) Percentage of EGFP, EGFP-LCA1, or EGFP-LC/A3 colocalized with WGA on the cell membrane was quantified. Ten random fields of EGFP-transfected cells were scored positive for membrane localization when EGFP colocalized with WGA on the cell membrane. The percent positive for membrane localization was scored as the was scored as the percent EGFP localized/(total number of EGFP-positive cells scored × 100), as previously described [40].

.

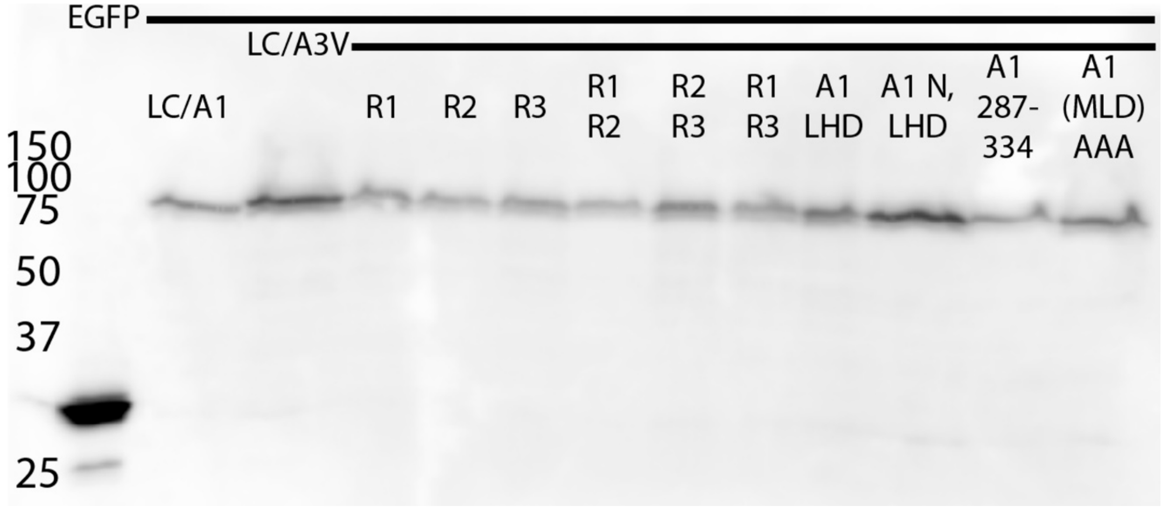

Supplemental Figure S2 Western blotting of GFP-LC/A3V (A1- LHD) chimeras. The protocol was utilized as described [241]. Cell lysates for N2A cells transfected the DNA encoding the indicated GFP-fusion protein were subjected to 13.5% SDS-PAGE, transferred to a PVDF membrane, and probed for GFP with rat-anti-EGFP antibody 1:3000 followed by Goat-anti-rat IgG-HRP (1:10000) with Super Signal for detection as described in the methods section. The GFP signal was detected in lysates expressing GFP-fusion protein ~75 kDa, while lysates expressing GFP alone yielded a (GFP) reactive band at ~26 kDa.

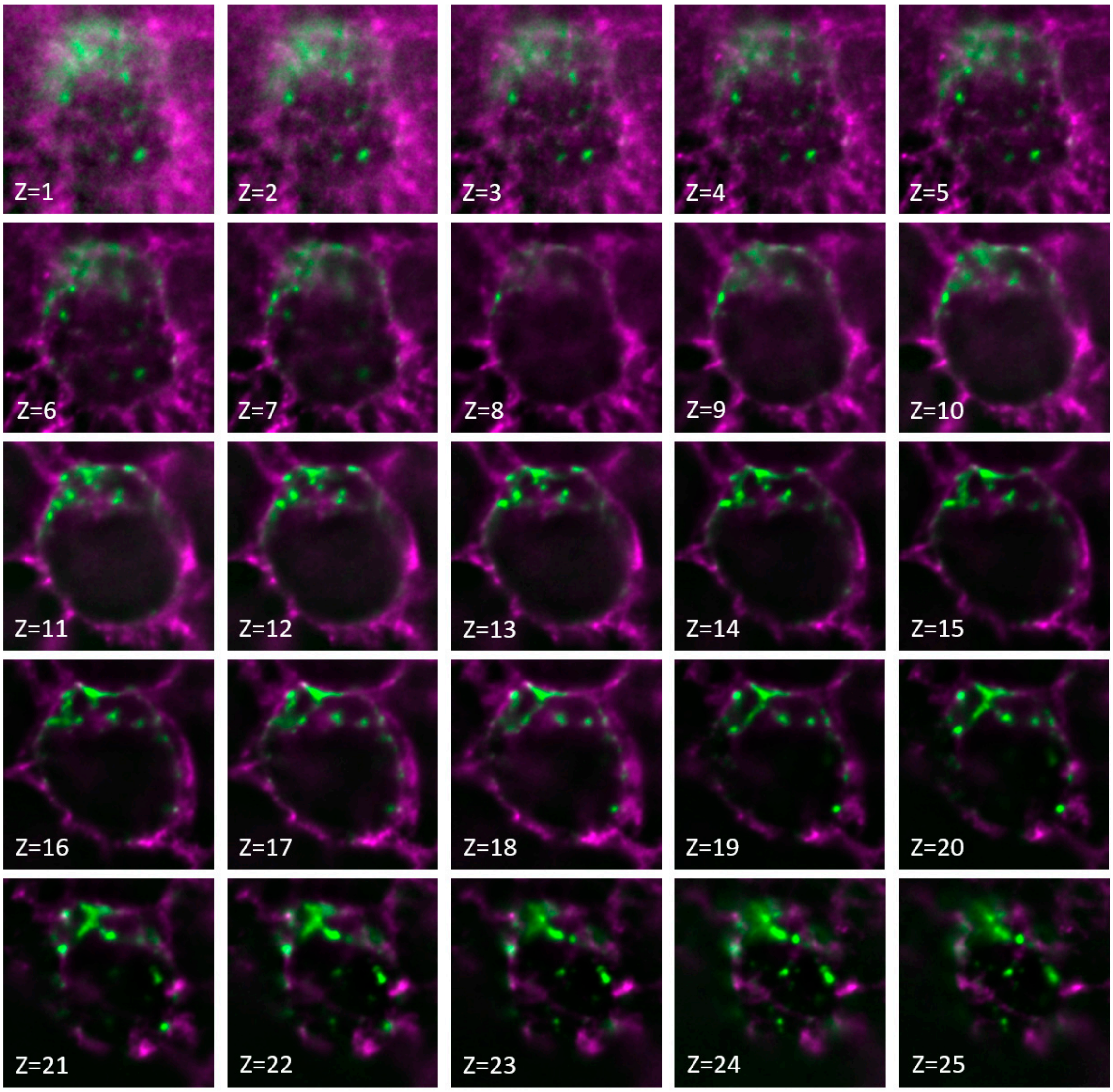

Supplemental Figure S3. Z-stack series of GFP-LC/A1 and SNAP-25. Neuro-2a cells were transfected with 500 ng of GFP-LC/A1 (green); Neuro-2a cells were stained for SNAP-25 (purple) after an overnight transfection. A visual examination of each Z-series shows that the signal from LC/A1 localizes with SNAP-25 at the plasma membrane, indicated by white. However, intracellular GFP-LC/A1 remains primarily segregated from SNAP-25, indicating that the trafficking of LC/A1 to the plasma membrane is uncoupled from SNAP-25. The compiled image was separated into 3 µm step increments representing each Z-series.

```
A3LM    ----------R---------------A------------EGV------------------   60
A1      MPFVNKQFNYKDPVNGVDIAYIKIPNVGQMQPVKAFKIHNKIWVIPERDTFTNPEEGDLN   60
        ****** ***:** ************.************: :******************

A3LM    -------------------------------I---D-----G------SF--K-------   120
A1      PPPEAKQVPVSYYDSTYLSTDNEKDNYLKGVTKLFERIYSTDLGRMLLTSIVRGIPFWGG   120
        ******************************* ***:*****.******: **:*******

A3LM    -------------C----E-G------------T----------C-----D---------   180
A1      STIDTELKVIDTNCINVIQPDGSYRSEELNLVIIGPSADIIQFECKSFGHEVLNLTRNGY   180
        ******************:*.************ ****************:*:*******

A3LM    -------------------------------T----------------A-----------   240
A1      GSTQYIRFSPDFTFGFEESLEVDTNPLLGAGKFATDPAVTLAHELIHAGHRLYGIAINPN   240
        *******************************.**************** ***********
                                              Region 1: 275-300
A3LM    --L-K-----------------------N-TNF----WQKK-SRDA-DNLQNI-RI--E-   300
A1      RVFKVNTNAYYEMSGLEVSFEELRTFGGHDAKFIDSLQENEFRLYYYNKFKDIASTLNKA   300
        **:**:**********************:*::***** :::*    *::::::*  **:*
              Region 2: 302-334                 Region 3: 335-357
A3LM    -T-----TP------I-IR--F----A---I--N-AA-KEF-RV--RGF--LE--NP---   360
A1      KSIVGTTASLQYMKNVFKEKYLLSEDTSGKFSVDKLKFDKLYKMLTEIYTEDNFVKFFKV   360
        *:*:***: ******:* .**:****:***:**:*  *.::*::**. :** :**: ***

A3LM    --------------R-----DE----NE----EG    --S--------SR---R-----   416
A1      LNRKTYLNFDKAVFKINIVPKVNYTIYDGFNLRNTNLAANFNGQNTEINNMNFTKLKNFT   420
        :*************:*****. **** :****..    :* ********. ***:*****

A3LM    ---------C-----PF------E------   446
A1      GLFEFYKLLCVRGIITSKTKSLDKGYNKAL   450
        *********.*****  ******:******
```

Supplemental Figure S4 Blastp alignment of LC/A3 LM with LC/A1. The primary amino acid sequences of A3LM (*Top*) ACA57525 and A1 (bottom) ACS66881 were analyzed by Blastp. The (*Bottom*) line depicts identical amino acids between A3 LM and A1 (*); conserved amino acids (:); and non-conserved amino acids ( ). Region-1 residues 275-300, region-2 302-334, and region-3 335-357 are boxed and labeled above each region, respectively.   Cysteine residues are bolded and underlined.
